# Avidity maturation of humoral response following primary and booster doses of BNT162b2 mRNA vaccine among nursing home residents and healthcare workers

**DOI:** 10.1007/s11357-024-01215-y

**Published:** 2024-05-25

**Authors:** Oladayo A. Oyebanji, Nicholas Sundheimer, Vaishnavi Ragavapuram, Brigid M. Wilson, Yasin Abul, Stefan Gravenstein, Jürgen Bosch, Christopher L. King, David H. Canaday

**Affiliations:** 1 Division of Infectious Diseases and HIV Medicine, Case Western Reserve University School of Medicine, Cleveland, OH USA; 2 Center for Global Health and Diseases, Case Western Reserve University, Cleveland, OH USA; 3 Geriatric Research Education and Clinical Center, Department of Veterans Affairs Medical Center, Louis Stokes Cleveland, Cleveland, OH USA; 4 Center of Innovation in Long-Term Services and Supports, Veterans Administration Medical Center, Providence, Rhode Island USA; 5 Brown University School of Public Health Center for Gerontology and Healthcare Research, Providence, Rhode Island USA; 6 Division of Geriatrics and Palliative Medicine, Alpert Medical School of Brown University, Providence, Rhode Island USA

**Keywords:** Avidity, Affinity maturation, Nursing home residents, Healthcare workers, COVID-19, Bivalent boosters, Omicron, BNT162b2 mRNA vaccine

## Abstract

Infections, despite vaccination, can be clinically consequential for frail nursing home residents (NHR). Poor vaccine-induced antibody quality may add risk for such subsequent infections and more severe disease. We assessed antibody binding avidity, as a surrogate for antibody quality, among NHR and healthcare workers (HCW). We longitudinally sampled 112 NHR and 52 HCWs who received the BNT162b2 mRNA vaccine after each dose up to the Wuhan-BA.4/5-based Omicron bivalent boosters. We quantified anti-spike, anti-receptor binding domain (RBD), and avidity levels to the ancestral Wuhan, Delta, and Omicron BA.1 & 4/5 strains. The primary vaccination series produced substantial anti-spike and RBD levels which were low in avidity against all strains tested. Antibody avidity progressively increased in the 6–8 months that followed. Avidity significantly increased after the 1st booster but not for subsequent boosters. This study underscores the importance of booster vaccination among NHR and HCWs. The 1st booster dose increases avidity, increasing vaccine-induced functional antibody. The higher cross-reactivity of higher avidity antibodies to other SARS-CoV-2 strains should translate to better protection from ever-evolving strains. Higher avidities may help explain how the vaccine’s protective effects persist despite waning antibody titers after each vaccine dose.

**Supplementary Information:**

The online version contains supplementary material available at 10.1007/s11357-024-01215-y.

## Introduction

Vaccine-induced immunity has mitigated Coronavirus disease 2019 (COVID-19), reducing morbidity and mortality to more modest rates, most dramatically for the frail nursing home (NH) population [1, 2]. Follow-up studies have demonstrated a significant decline in humoral immunity over the months following vaccination, making the case for booster doses [3, 4]. Despite the significant immunologic and clinical benefits associated with the boosters [5–8], the emergence of the Omicron variant resulted in a dramatic increase in infections, even among vaccinees who completed the primary and booster series, and notably in frail and vulnerable NH populations [9].

Establishing definitive immunologic correlates of protection in the face of rapidly developing variants has remained elusive, yet increased binding and neutralizing antibody titers are associated with protection against symptomatic SARS-CoV-2 infections [10, 11]. The SARS-CoV-2 virus’ spike protein (S) facilitates entry into cells via the receptor-binding domain (RBD) by binding to the human angiotensin-converting enzyme 2 (ACE-2) receptor [12]. The avidity of RBD binding would be predicted to translate to the quality of protection, and offer a better measure than just antibody quantification for assessing vaccine-induced immunity and determining correlates of effective protection.

Antibody avidity is the total binding strength of an antibody to its specific epitope and is a consequence of the overall maturation of the humoral response [13–15]. It progressively increases after antigenic stimulation by infection or vaccination and occurs due to affinity maturation [16–18]. Avidity can thus help distinguish recent from more remote infections, as acute infections produce low avidity antibodies that typically mature progressively over time [19, 20]. Like other seasonal coronaviruses, SARS-CoV-2 infection produces antibodies with low and intermediate avidity titers that plateau early [21, 22]. In contrast, repeated doses of the BNT162b2 mRNA vaccination produced highly avid antibodies that bound more variants in convalescent and infection-naive subjects highlighting the qualitative benefit of repeat vaccinations [23–25].

While neutralizing antibodies helps assess antibodies qualitatively, we know little about the effective binding strength of these antibodies over time. As vaccine-induced antibodies wane quantitatively, it is thus critical to assess the effect of this longitudinal decline on the strength of their binding abilities. We have previously reported on the kinetics of binding and neutralization antibodies in this cohort of NH residents (NHRs) and healthcare workers (HCWs) [26–29]. Here, we extend our study of this cohort to examine the kinetics of avidity maturation as a surrogate for effective antibody binding, from the initial BNT162b2 mRNA primary vaccination series through the administration of the booster doses among NHRs and HCWs.

## Methods

### Participants' demographics and sampling

Study approval was obtained from the WCG institutional review board. All participants or their legally authorized representatives provided informed consent. NHRs and HCWs were sampled from 3 community NHs and one state Veterans Home. Additionally, HCWs that included hospital and laboratory staff were recruited from the Cleveland Department of Veterans Affairs Medical Center and Case Western Reserve University. All sites administered the BNT162b2 mRNA vaccine primary series in December 2020 and January 2021 followed by a second dose 3 weeks later during the emergency use authorization period, then a monovalent first booster dose (3rd dose) 6 months or longer after their primary series and a second monovalent booster (4th dose) 6 months or longer after the 1st booster and then a bivalent booster (5th dose). Not all participants received every booster dose.

Participants were deemed to have a “*prior infection*” if they had a known history of SARS-CoV-2 infection confirmed by PCR or antigen test, and/or elevated antibody levels to SARS-CoV-2 nucleocapsid (N) protein detected from serum collected at each timepoint. Otherwise, participants were classified as “*infection-naive.*” Throughout the longitudinal study, samples from participants who had laboratory-confirmed–PCR test, antigen test, and/or immunological (anti-N-protein)–evidence of infection after vaccination were excluded from the analysis at the timepoint immediately following the laboratory-confirmed SARS-CoV-2 infection. Once such an infection was detected, data from subsequent samples were re-classified as belonging to subjects who had “prior” infection.

**Fig. 1:**
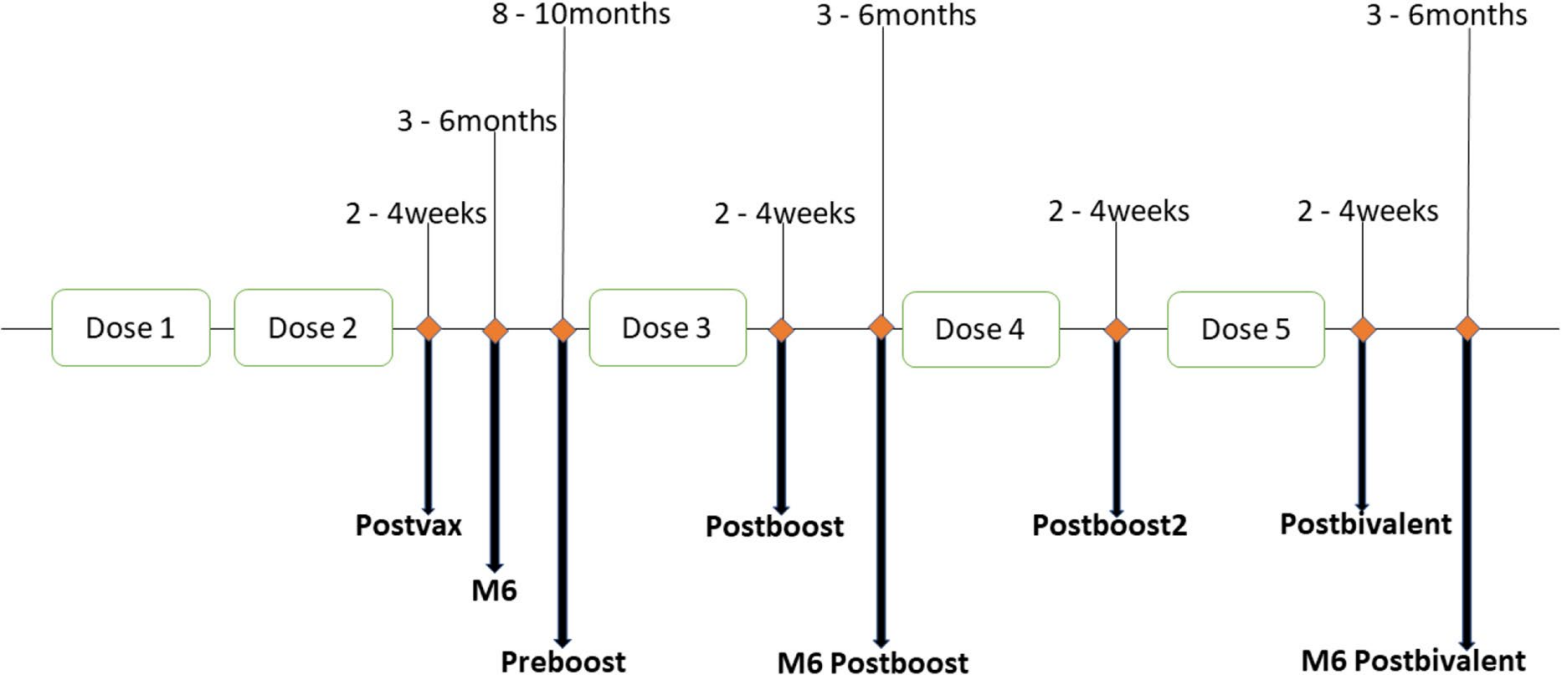
Timeline of blood sampling from participants. Serum samples were collected from participants at different time points after BNT162b2 mRNA vaccination. Doses 1 and 2 are the 1st and 2nd doses, respectively, given 3 weeks apart. Doses 3 and 4 are the 1st and 2nd monovalent booster doses, given at least 6 months after the previous dose. Dose 5 is a Wuhan-Omicron BA.4/5-containing bivalent booster dose. While many of our participants did not receive the 2nd monovalent booster (Dose 4), those who did, got the bivalent booster within 4-6 months

Serum samples were obtained at the following time points: 2–4 weeks and 6 months after completion of the primary series; 0–14 days before (generally 7–9 months after the primary series), 2–4 weeks and 3–6 months after the 1st monovalent booster; 2–4 weeks and 3–6 months after the 2nd monovalent booster; 2–4 weeks and 3–6 months after the bivalent booster (Fig. 1).

### Anti-spike and anti-RBD assay

**Fig. 2:**
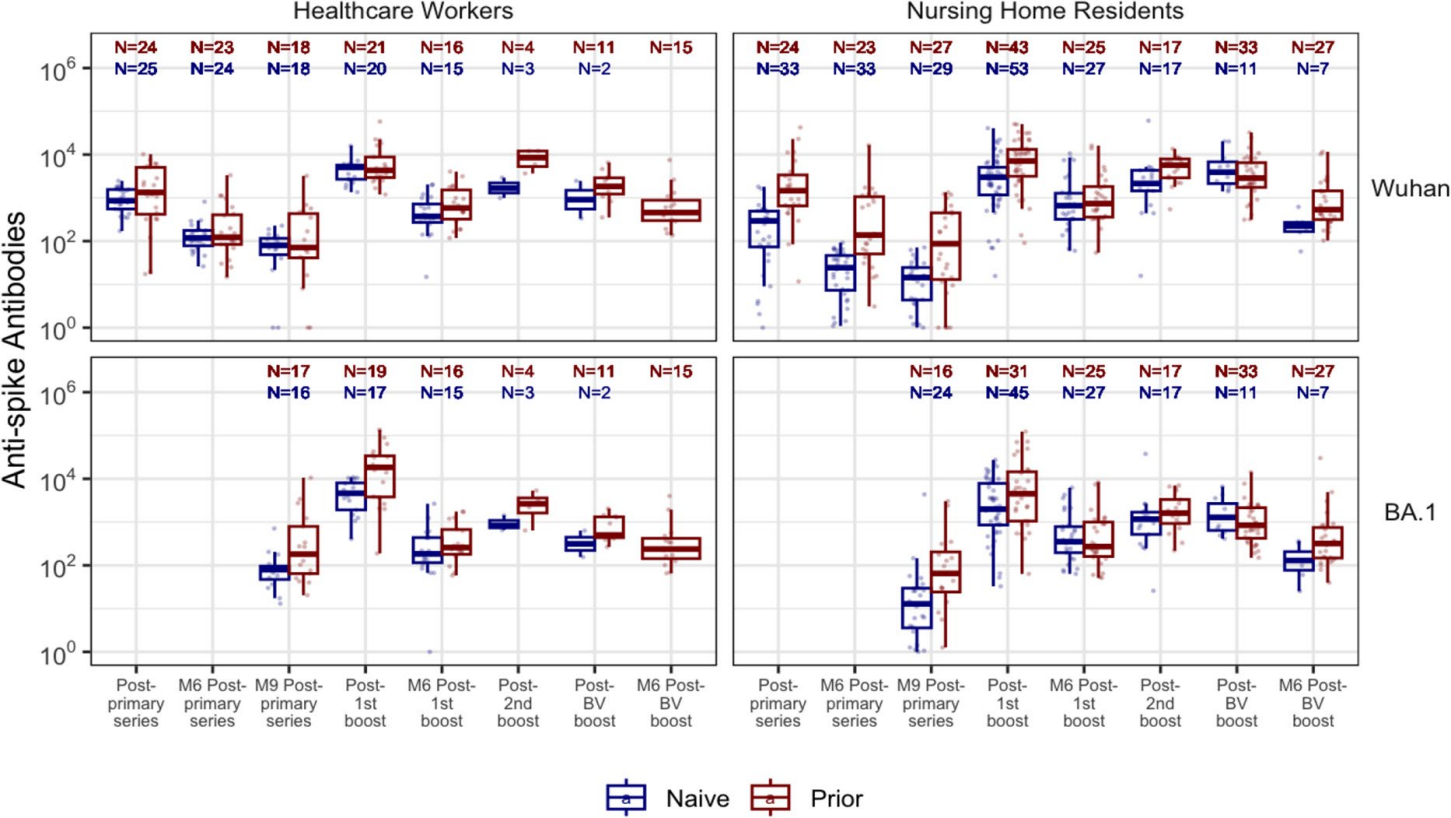
Spike Antibody titers over time (Wuhan & BA.1) - nursing home residents & healthcare workers. The figure shows the kinetics of anti-spike antibodies against the Wuhan and Omicron BA.1 strains across different time points among nursing home residents and healthcare workers. Wuhan anti-Spike is measured in BAU/ml while BA.1 is measured in AU/ml. Boxplots show medians (middle line), and third and first quartiles (boxes), while the whiskers display the minimum and maximum values. Post-vaccination sera were taken 2-4 weeks after each dose while M6 and M9 sera were taken 6-8 months and 7-10 months later. Blue: Naive subjects, Red: Prior subjects. BV: Wuhan-Omicron BA.5 Bivalent booster

**Fig. 3:**
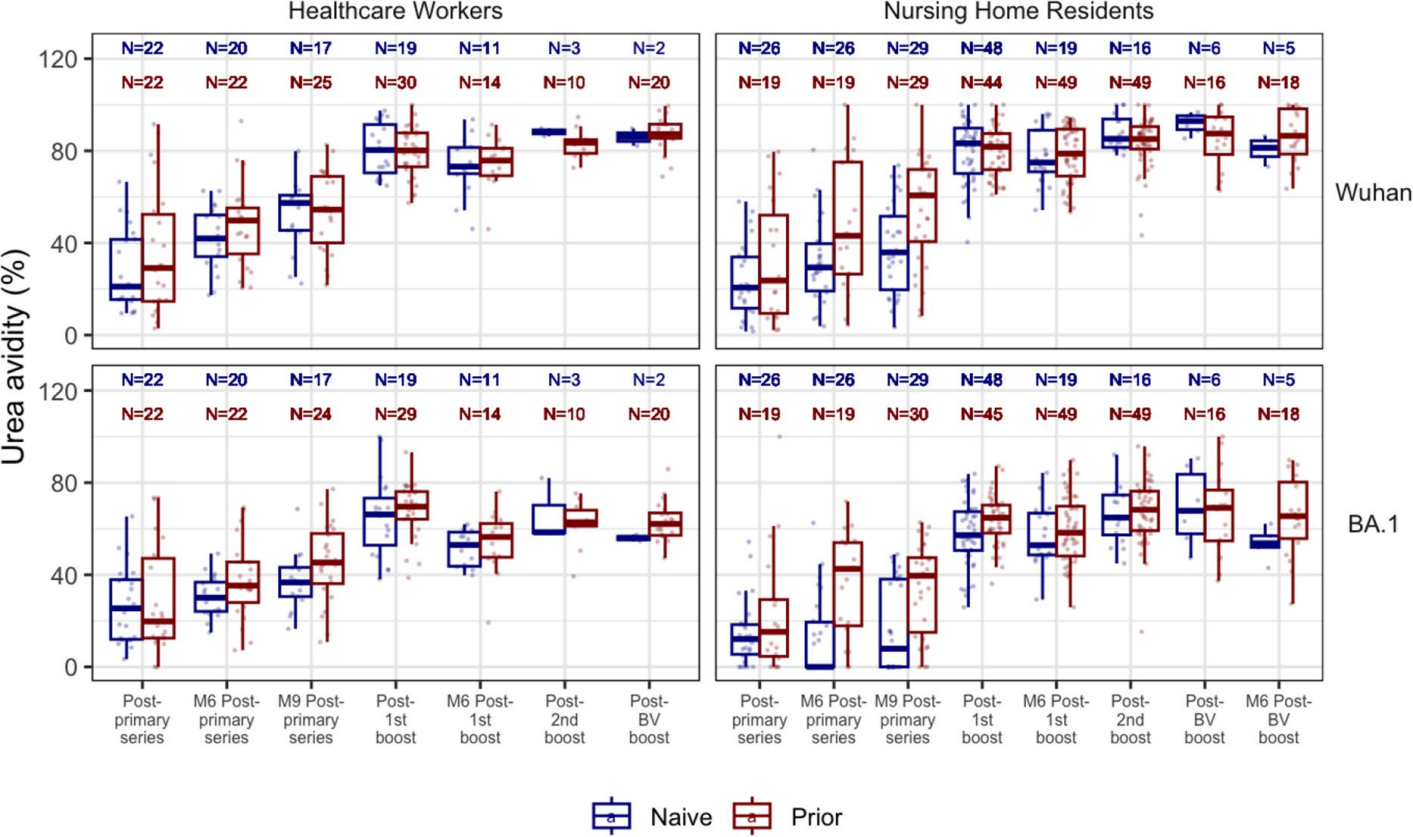
Avidity of anti-spike IgG against Wuhan and Omicron BA.1 strains over time. The figure shows the relative avidity of anti-spike antibodies against the Wuhan and Omicron BA.1 strains across different time points among nursing home residents and healthcare workers measured using 6M urea as a chaotropic reagent. Antibody avidity was expressed as avidity index in %. Boxplots show medians (middle line) and third and first quartiles (boxes), while the whiskers display the minimum and maximum values. Post-vaccination sera were taken 2-4 weeks after each dose, while M6 and M9 sera were taken 6-8 months and 7-10 months later. Blue: Naive subjects (no prior infection), Red: Prior subjects (previously infected). BV: Wuhan-Omicron BA.5 Bivalent booster

Immune response to the vaccine was assessed using a bead-multiplex immunoassay using Wuhan, Delta, and Omicron BA.1, 4/5 strains [28]. Anti-spike IgG (S) generated a result of BAU/ml based on the Frederick National Laboratory standard, which was calibrated to the WHO 20/136 standard, and the anti-receptor binding domain (RBD) generated a result in arbitrary units (AU). Stabilized full-length S protein (aa 16–1230, with furin site mutated and recombinant SARS-CoV-2 S(1–1208)-2P-3C-His8-TwinStrep) and RBD (aa 319–541) were conjugated to magnetic microbeads (Luminex) and Magpix assay system (BioRad, Inc). The mean fluorescent index was recorded after detecting antigen-specific IgG in participant serum using PE-conjugated Donkey F(ab)2 anti-human IgG, with Fcγ (Jackson ImmunoResearch, Grove, West Grove, PA). Figs. 2, 3

### Determination of avidity

The MagPix assay system was used to generate MFI (mean fluorescence intensity) values, generating numerical data that measured the fluorescent intensity of antibodies (IgG) bound to their specific antigen (anti-spike and anti-RBD: Wuhan, BA.1, and BA.4/5). Patient serum samples were subjected to a normal assay buffer (1 × PBS, 10% BSA, 0.01% Tween20), and to measure avidity, two different conditions were established using the same bead-multiplex immunoassay and MFI measurement strategy: samples were tested with and without the presence of 6 M Urea. Plates exposed to 6 M urea were incubated for 30 min at room temperature, with the first 10 min on a plate shaker. The relative avidity index (AI) was calculated as the percentage difference between the MFI values in the presence of 6 M urea divided by the MFI values in the absence of that chaotropic agent [30].

**Fig. 4:**
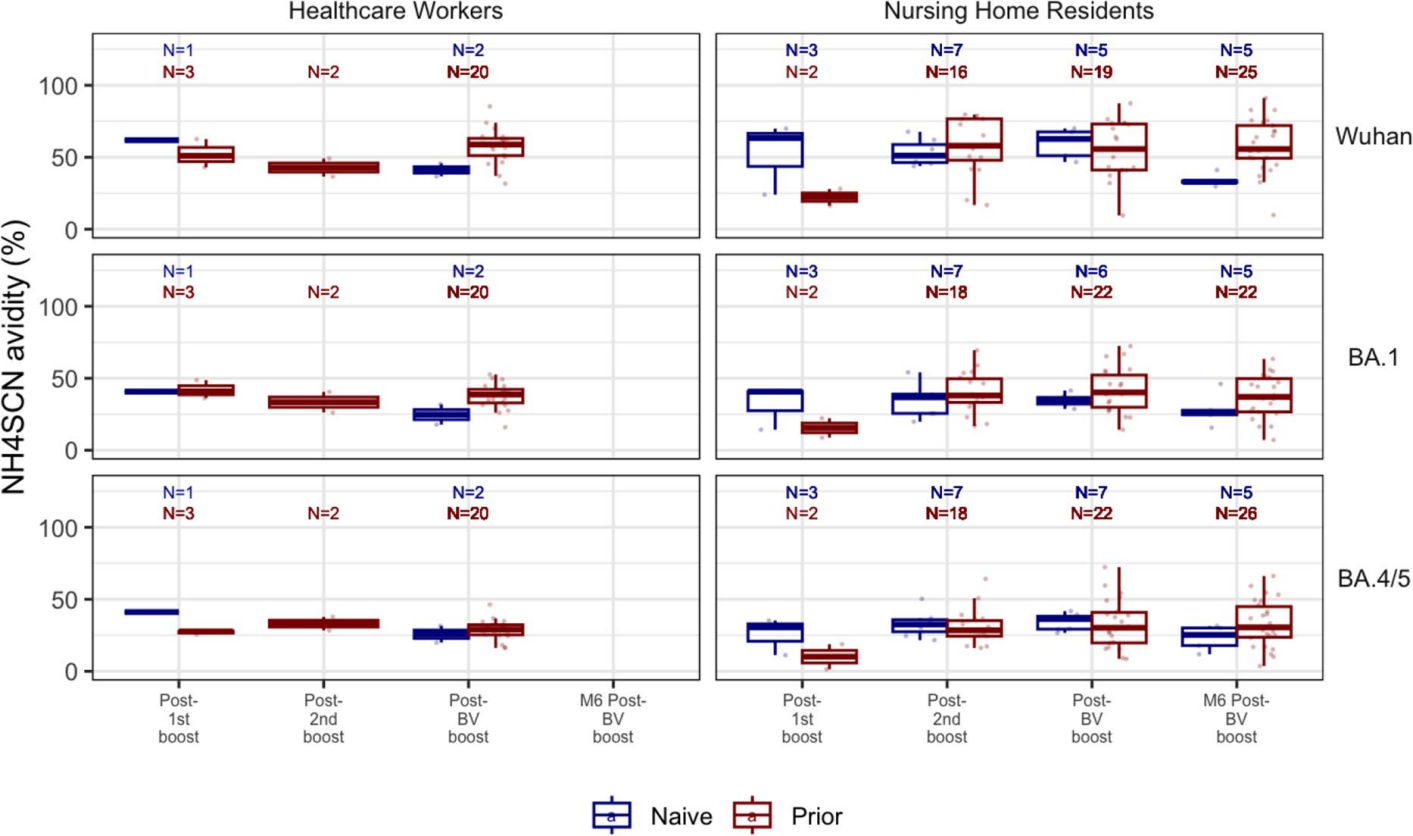
Spike avidity determined by 2.1 M NH_4_SCN across the booster doses. Relative avidity of anti-spike antibodies against the Wuhan, Omicron BA.1, and BA.5 strains across different time points among nursing home residents and healthcare workers was measured using ammonium isothiocyanate (NH_4_SCN). Antibody avidity is expressed as avidity index in %. Boxplots show medians (middle line), and third and first quartiles (boxes), while the whiskers display the minimum and maximum values. Post-vaccination sera were taken 2-4 weeks after each booster dose, while M6 post-BV serum was obtained 6-8 months after the BV Booster dose. Blue: Naive subjects (no prior infection), Red: Prior subjects (previously infected). BV: Wuhan-Omicron BA.5 Bivalent booster

In addition, a subset of serum samples across the booster groups was exposed to 2.1 M ammonium isothiocyanate (NH_4_SCN) treatment to characterize their binding avidity further as the NH_4_SCN was found to be a more stringent chaotropic agent. This was done using the same bead-multiplex immunoassay and MFI measurement strategy that determined the avidity of 6 M urea. The concentration of 2.1 M was chosen after exposing samples to 11 different concentrations ranging from 0 to 4.0 M. Instead of measuring avidity using AI, half maximal inhibitory concentration (IC_50_) calculations were made to determine the approximate concentration of all samples where the antibody binding was reduced by half. Incubation times differed from using 6 M urea; samples were incubated for 20 min at room temperature, with the first 10 min on a plate shaker (Fig. 4).

The criteria for assessing antibody avidity were arbitrarily set based on the following ranges: < 30% (low avidity), 30–59% (intermediate avidity), 60–75% (high avidity), and > 75% (very high avidity) [19, 31].

### Statistical analysis

For each blood sampling time, all available urea and NH_4_SCN avidity, anti-Spike, and anti-RBD antibody assay data were summarized for each tested strain after stratifying subjects by cohort (NHR or HCW) and prior infection at the given time. All subject-level results and group summaries were presented graphically, and the median and interquartile range were calculated within assay, strain, and subject group and presented for times and subject groups with at least 5 observations. For urea avidity, these median values were used to classify groups as low avidity (< 30%), intermediate avidity (30–59%), high avidity (60–75%), and very high avidity (> 75%) across time.

To statistically compare urea avidity over time and between subject groups, linear mixed-effects models were estimated in which a random intercept was estimated for each subject to adjust for repeated measures. Urea avidity was predicted with sampling time, subject cohort, and infection status, including interaction effects. Post-hoc contrasts between groups and/or sampling times were generated when significant effects were detected to characterize the effect. All analyses were performed in R Version 4.2.2. Models and contrasts were estimated using functions with the nlme and emmeans packages.

## Results

This analysis reports on sera obtained from 164 subjects comprising 112 NHRs and 52 HCWs. The median age for NHRs is 76 (70,85), with 62% Male while HCWs have a median age of 52 with 52% female (44,58). The detailed characteristics of these subjects are presented in Table 1.

**Table 1:** Baseline demographics of participants

	Healthcare Workers	Nursing Home Residents
Age: Med (IQR)	52 (44,58)	76 (70,85)
Age: Mean (SD)	51 (10)	77 (11)
Age: Range	31–68	51–100
Male	25 (48%)	70 (62%)
Female	27 (52%)	42 (38%)
Race: White	43 (83%)	91 (81%)
Race: Black	3 (6%)	20 (18%)
Race: Hispanic	2 (4%)	1 (1%)
Race: Asian	3 (6%)	0 (0%)

*IQR* Interquartile range, *SD* Standard deviation

### The first two doses of the BNT162b2 mRNA vaccine result in initially low avidity antibodies

**Fig 5:**
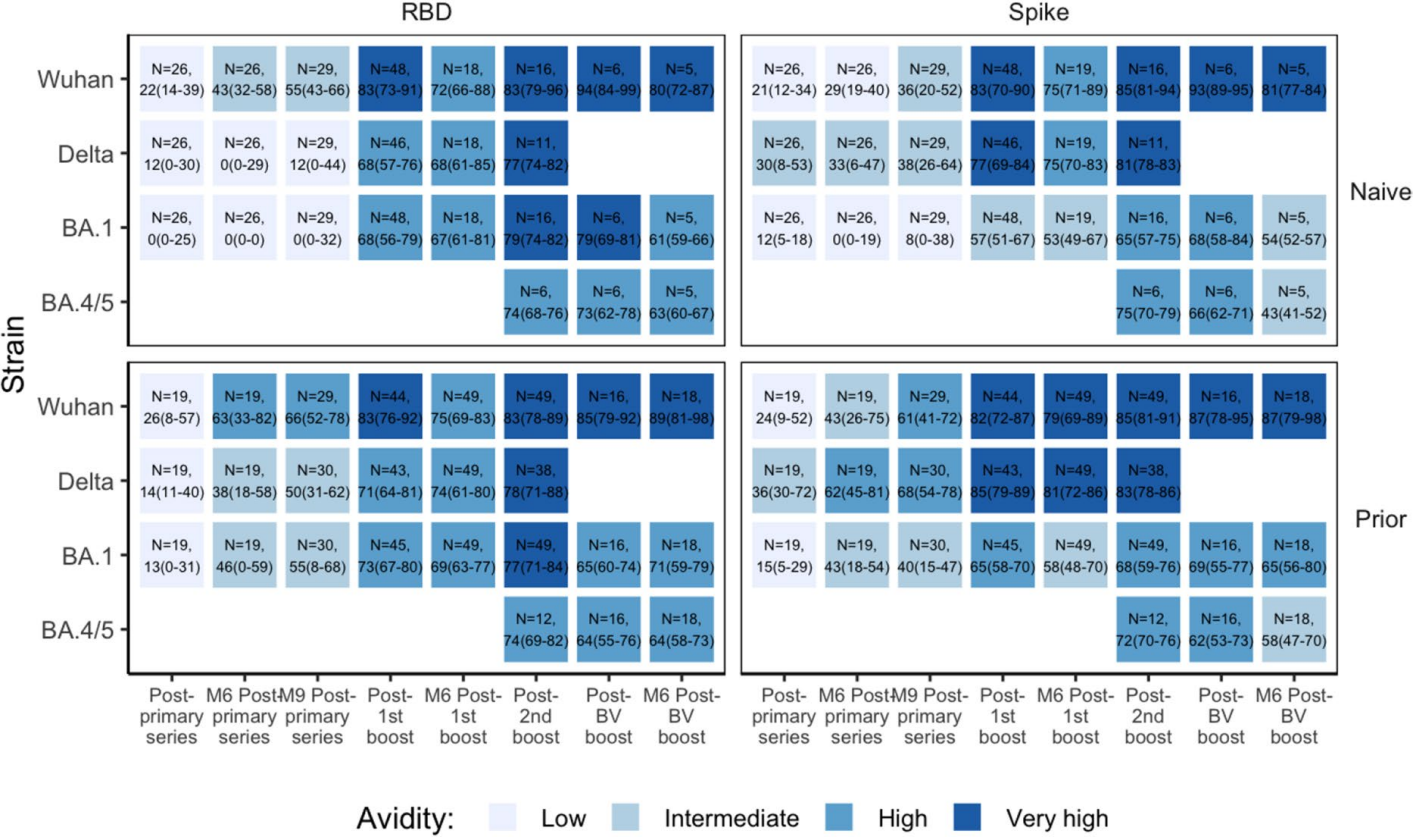
Relative anti-S and anti-RBD avidity index by strain, and prior infection status among nursing home residents. This figure, color-coded according to avidity level, presents the median avidity values (with interquartile ranges) measured using 6M Urea. Postvaccination sera were taken 2-4 weeks after each dose while M6 and M9 sera were taken 6-8 months and 7-10 months later. Avidity is expressed as avidity index in % and assessed based on the following ranges: <30% (low avidity), 30-59% (intermediate avidity), 60-75% (high avidity), and >75% (very high avidity). Naive subjects: no prior infection), Prior subjects: previously infected. RBD: Receptor-Binding Domain

Initial avidity levels following the primary 2 doses of BNT162b2 mRNA vaccination in all groups show the production of antibodies with low avidity < 30% to both S and RBD across all strains– Wuhan-Hu-1, Delta, and Omicron BA.1. Despite a substantial decline in COVID-19-specific IgG levels over time (Figs. 2, 3 & S3), the Wuhan S avidity increases over the 6–9 months following the primary vaccination for all groups, except the NHR naive group, which rises to an intermediate level (30%-50%) (Fig. 3,5). Although the NHR naive group also demonstrates an increase in avidity, they remained in the low range. Earlier, we reported that this group had the lowest response to the 2-dose BNT162b2 primary vaccination series relative to its comparator HCW and individuals with prior infection [26]. Except for the NHR naive group, which plateaus in the intermediate range, maturation of the Wuhan anti-RBD avidity generally occurs faster than that of the anti-S as all groups progress to the intermediate and then to the highly avid range (Fig. S3).

Omicron BA.1 IgG levels to S are low following the primary series, but avidity increases to the intermediate levels except for the two naive groups—NHR and HCW, whose avidity remains low despite antibody level increases (Fig. 3). The BA.1 RBD avidity pattern is similar to that of Wuhan; however, its avidity rapidly increases in all groups from intermediate levels to highly avid levels 6–9 months following the second dose of the primary series (Fig. S3).

### Monovalent BNT162b2 mRNA boosters produce highly avid anti-S & anti-RBD antibodies against Wuhan, *Delta*, and Omicron BA.1

The 1st booster significantly elevates S and RBD IgG levels to Wuhan and Omicron BA.1 (Fig. 2, S1). This increase in IgG is matched by increased avidity levels in all groups examined. Wuhan S and RBD antibodies demonstrated very high levels of avidity in all groups (> 75%) (Fig. 3, 5, S3). This increase is particularly dramatic among the NHR naive group, whose antibodies remain in the low avidity range after the primary vaccine series. All groups except the NHR naive show a slight reduction in anti-S and anti-RBD avidity 3–6 months after the 1st booster. However, avidity remains high (> 60%). The 2nd booster restores the modestly declining avidity levels for all groups except the NHR naive group, extending the high avidity plateau that begins with the 1st booster.

Omicron BA.1 S and RBD avidity are elevated in all groups following receipt of the 1st monovalent booster except the NHR naive group, whose anti-S avidity index is slightly below the high avidity range. Their avidity progressively increases to develop highly avid antibodies 3–6 months later and more after the 2nd monovalent booster. The other groups slightly dropped their anti-S avidity level to the intermediate range, but the 2nd booster restored high avidity levels. Anti-RBD avidity remains in the high range for all groups, with the NH naive groups showing ongoing maturation.

### Avidity plateau formed across booster doses

Subsequent boosters induce no increase in avidity. Each extra booster dose produced highly avid antibodies but no higher levels than that attained after the 1st monovalent booster. Even the bivalent boosters do not increase avidity to the new antigen BA.4/5 spike (Fig. 4). We see this across all strains in both NH and HCW groups (Fig. 3, S2, S3). To further characterize this plateau response across the booster groups, the application of the stronger chaotropic agent, sodium isothiocyanate (NH_4_SCN), leads to a greater dissociation of the antigen–antibody complex, generating relatively lower-avidity values but shows a similar plateau pattern across the boosters with no significant difference (Fig. 4).

Using a linear mixed-effects model was estimated to compare Wuhan urea avidity across time with possible interactions of cohort and infection status, no cohort effect was detected. With post-hoc contrasts comparing infection naive to prior infection at each time, avidity differed significantly (p < 0.05) for all pre-boost sampling times, while no differences were detected at any post-boost sampling times. Comparing paired sampling times within infection status, we found that all pre-boost sampling points differed from all post-boost sampling times (p < 0.05 for all) and no differences were detected among the post-boost sampling times, further demonstrating the plateau of avidity following doses beyond primary series. (Figures S4 and S5).

In summary, the primary 2 doses of the BNT162b2 mRNA vaccine increase anti-S and RBD IgG with only low-avidity antibodies initially. However, avidity increases in 6–9 months despite waning anti-S and RBD IgG levels. The 1st monovalent booster restores waning antibody levels and produces high avidity antibodies. These high-avidity antibodies persist following subsequent booster doses, including the bivalent booster. Notably, further boosters do not result in any further increases in avidity.

## Discussion

We found that both nursing home residents (NHRs) and healthcare workers (HCWs) did not produce much high avidity antibody on first primary series antigen exposure, but with time, infection and/or additional boosters, almost all participants’ antibody avidity eventually improved and produced some cross-strain binding. Although antibody avidity increases progressively following initial antigenic exposure, incomplete avidity maturation has been noted with those generated against SARS-CoV-2 infection, evident by the low and intermediate avidity index which persists months after infection [21, 22]. In addition to immune-evading mutant strains, this incomplete affinity maturation of SARS-CoV-2-induced antibodies matched with waning antibody levels may produce a window of vulnerability to infection or re-infection in the several months after the initial vaccination. This contrasts with that reported for other viruses where once the infection becomes established, avidity progressively increases through affinity maturation until they become highly avid [19, 20]. However, the observation with the primary series COVID-19 vaccination of an enhanced maturation process in healthcare workers demonstrates a significant increase in their avidity index [23]. In this study, we report the kinetics of avidity maturation following 5 doses of the BNT162b2 mRNA vaccine among both previously infected and infection-naive NHRs and HCWs.

The primary vaccination series initially produced low-avidity antibodies despite the induction of substantial antibody levels. This observation contrasts with that reported by Struck, et. al., and Rastawicki et. al., where high avidity antibodies appeared about 3 weeks after the 2nd dose of the BNT162b2 mRNA vaccine [32, 33]. We speculate that differences in the population studied, avidity cutoffs and chaotropic agents used may account for the discordance from our findings. However, our findings do align with established observations where low-avidity antibodies are produced by recent antigenic exposure either via natural infection or vaccination [16, 19, 34, 35]. Antigen from infection and vaccination stimulates B cells to secrete antibodies and mature into memory B cells that can further differentiate into plasma cells on re-exposure to the antigen. Plasma cells sustain the humoral response following vaccination and infection. These two B cell lineages play distinct roles in humoral immunity's short- and long-term maturation. Transient plasmablasts are produced initially following each antigenic stimulation by an infection or vaccination [36, 37]. Thus, the rapid burst of antibodies produced by two vaccine doses is short-lived, as seen in the rapid waning of binding and neutralizing antibody titers evident as early as 3 months post-vaccination [38], while undergoing the affinity maturation process which is then expected to progress over time.

Interestingly, our findings further revealed a progressive increase in avidity months after the primary vaccine series. The mechanism that guides affinity maturation requires a prolonged and optimal supply of target antigens for a progressive increase in overall avidity levels [31, 39]. Thus, this finding of a progressive increase in avidity levels over time suggests an ongoing maturation process, supporting the observed persistent activation of B-cell germinal centers following mRNA vaccination [40, 41]. This progressive increase in avidity also occurs following exposure to SARS-CoV-2 infection. In their study of adults of different age groups, Pichler, et. al., observed a marked increase in avidity up to 7 months after confirmed infection with SARS-CoV-2 regardless of age, in line with reports from other studies [14, 42]. Wang, et. al., reported affinity maturation and persistence of SARS-CoV-2 specific B cells after the resolution of SARS-CoV-2 infection and dropping antibody levels [43]. However, they subsequently showed evidence of a persisting virus in the gut, driving further B cell maturation [44]. Thus, live attenuated vaccines or natural infection in naive individuals have persistence of antigens that allow ongoing affinity maturation. To explain the slowly–over months–progressive affinity maturation after mRNA vaccination, we speculate that mRNA vaccines may be more like natural infections or live attenuated vaccines than protein-based vaccines, because they induce protein production over time, unlike protein-based vaccines which deliver a one-time antigen load [41, 44, 45]. Additional research needs to confirm whether avidity maturation following a protein-based vaccine differs for this reason from that following an mRNA vaccine.

In our study, the 1st monovalent booster resulted in substantial antibody levels with high avidity in all groups regardless of prior infection status. This underscores the significant benefit of a COVID-19 booster vaccination in all populations. Some studies show individuals with high-avidity antibodies have a longer interval between vaccination and breakthrough infection than those with low-avidity antibodies [18, 34, 46]. This observation implies that avidity could serve as another surrogate, like neutralizing antibodies, for the duration of protection post-vaccination [10, 11, 13, 22]. However, neutralizing antibodies in this population declined in our population [8, 27, 38] while avidity rose, indicating that neutralizing antibodies alone do not capture all aspects of immunity important for protection from severe infection or its consequences. Protection from high-avidity antibodies may help offset that loss from declining IgG levels months after vaccination as much as high IgG levels may compensate for low-avidity antibodies generated early after vaccination [31]. Furthermore, this increase in avidity occurred across all the strains tested. With SARS-CoV-2 being notorious for ever-emerging immune-evasive strains, a hope for the original monovalent vaccines remains protection through cross-reactivity. In a previous study, we reported the significant production of binding and neutralizing antibodies against the Omicron BA.1 variant by a booster dose of the monovalent Wuhan-based BNT162b2 mRNA vaccine [8]. In the present studies, the 1st and 2nd Wuhan-based monovalent BNT162b2 mRNA boosters significantly produced antibodies of high avidity index to the Delta and Omicron BA.1 strains, similar to the findings of Dapporto, et. al., in their study of post-mRNA vaccine recipients [35].

Furthermore, we observed a saturation effect in the avidity index across the booster doses, including the bivalent booster, already apparent in the prior infected groups. This may imply infection helps complete affinity maturation with the 1st booster. This observation aligns with studies that reported generating maximum avidity levels by the 1st mRNA vaccine booster dose [33] and that subsequent booster doses do not necessarily increase the breadth of the humoral response [47].

Our interpretation has limitations. First, a comparative study of a similar longitudinal follow-up of non-vaccinated convalescent subjects would add context to the findings in this study [32]. Nonetheless, vaccine-induced antibodies have been predicted to confer better protection against variants than comparable levels of infection-generated antibodies [24]. Secondly, avidity has been reported to be a predictor and correlates with the severity of COVID-19 [39, 48]. We did not assess such associations. Lastly, BA.1 and BA.4/5 immunity may not confer protection against other Omicron subvariants [49]. Thus, we still need avidity studies involving antibodies generated against other Omicron subvariants.

To our knowledge, this is the first study detailing the longitudinal kinetics of avidity maturation following vaccination from the primary series through 2 monovalent booster doses up to the bivalent doses in any age group. The observation of cross-variant highly avid antibodies by the monovalent booster doses underscores the importance of additional doses in this vulnerable population. Together with the significant impact of the 1st booster on the quality and breadth of antibody response, a 3-dose primary regimen could be more beneficial and should be given early consideration in future pandemics involving a neoantigen [50].

## Supplementary Information

Below is the link to the electronic supplementary material.

Supplementary file1 (DOCX 817 KB)

## Data Availability

The de-identified dataset and related codes for analysis will be made available to researchers upon request after publication. Requests for data should be addressed to the corresponding author.
