# Supplementary material for: Avidity maturation of humoral response following primary and booster doses of BNT162b2 mRNA vaccine among nursing home residents and healthcare workers
Source: GeroScience. 2024 May 25;46(6):6183–94. doi: 10.1007/s11357-024-01215-y (PMC11493945; doi:10.1007/s11357-024-01215-y)
Supplement: Supplementary file 1 — Supplementary file1 (DOCX 817 KB) [file 11357_2024_1215_MOESM1_ESM.docx]

**Supplementary Figures.**


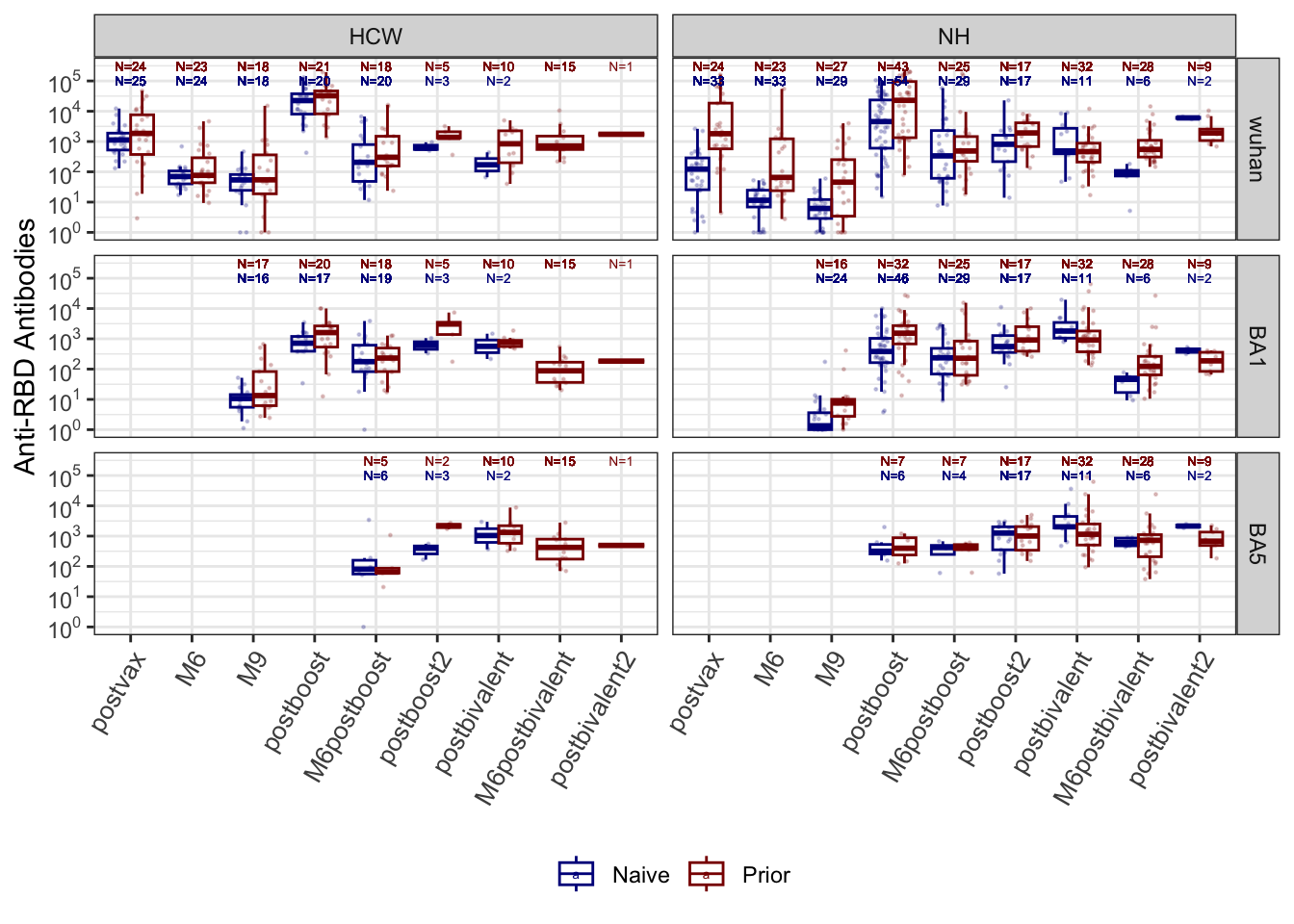


Fig. S1. **Anti-Receptor-Binding Domain (RBD) titers over time (Wuhan, BA.1 & BA.5) - nursing home residents (NH) & healthcare workers (HCW).** The figure shows the kinetics of anti-RBD antibodies against the Wuhan, Omicron BA.1, and BA.5 strains across different time points among nursing home residents and healthcare workers. Wuhan anti-RBD, BA.1, and BA.5 are measured in AU/ml. Boxplots show medians (middle line), and third and first quartiles (boxes), while the whiskers display the minimum and maximum values. Post-vaccination sera were taken 2-4 weeks after each dose while M6 and M9 sera were taken 6-8 months and 7-10 months later, respectively. Blue: Naive subjects, Red: Prior subjects. BV: Wuhan-Omicron BA.5 Bivalent booster.


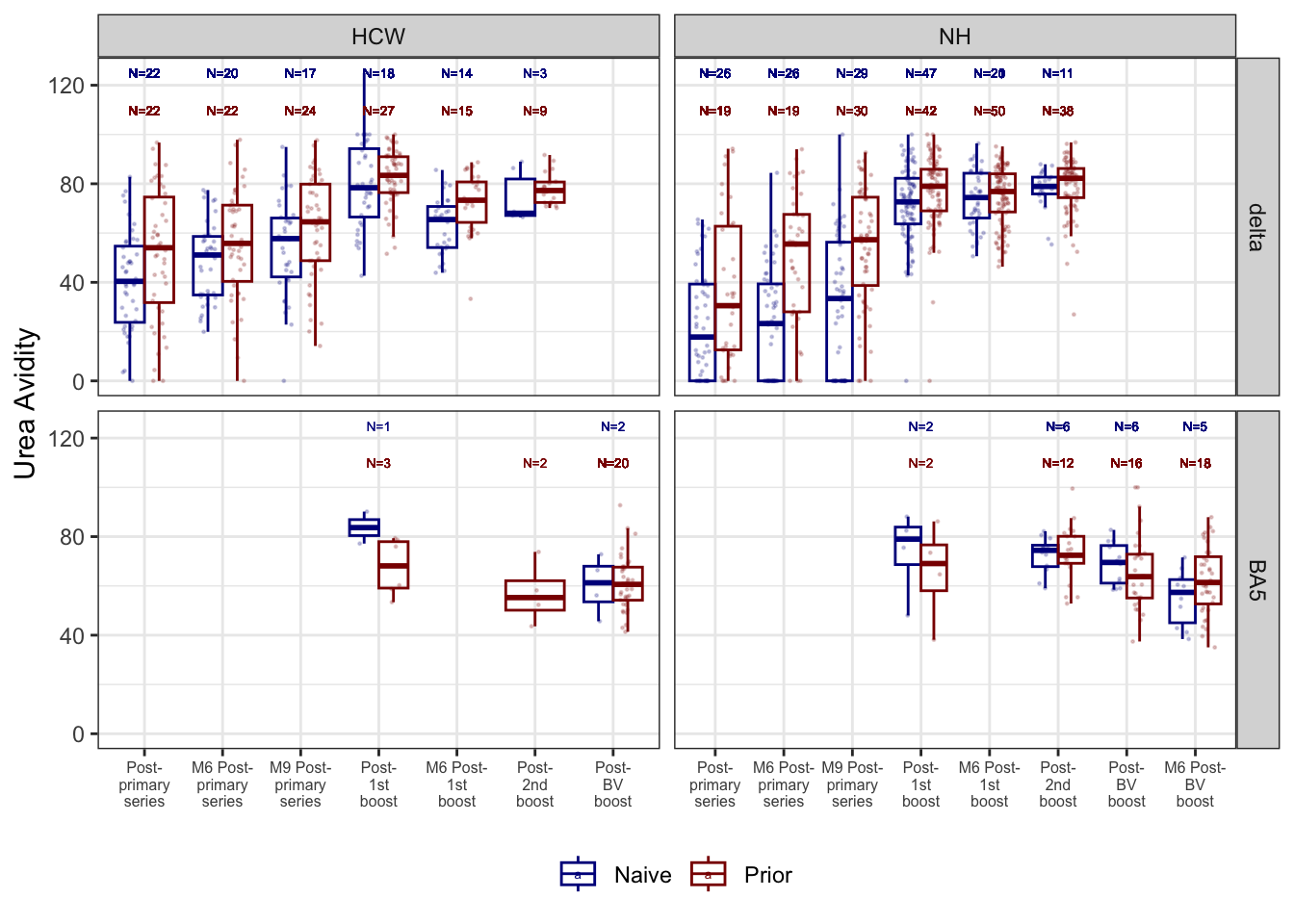


Fig. S2. **Anti-spike avidity over time (Delta & BA.5) - nursing home residents (NH) & healthcare workers (HCW).** The figure shows the kinetics of anti-spike avidity against the Delta and BA.5 strains across different time points among nursing home residents and healthcare workers. Avidity is measured by ELISA using 6M urea and expressed as avidity index in percentage (%). Boxplots show medians (middle line), and third and first quartiles (boxes), while the whiskers display the minimum and maximum values. Post-vaccination sera were taken 2-4 weeks after each dose while M6 and M9 sera were taken 6-8 months and 7-10 months later, respectively. Blue: Naive (no prior infection) subjects, Red: Prior (previously infected) subjects. BV: Wuhan-Omicron BA.5 Bivalent booster.


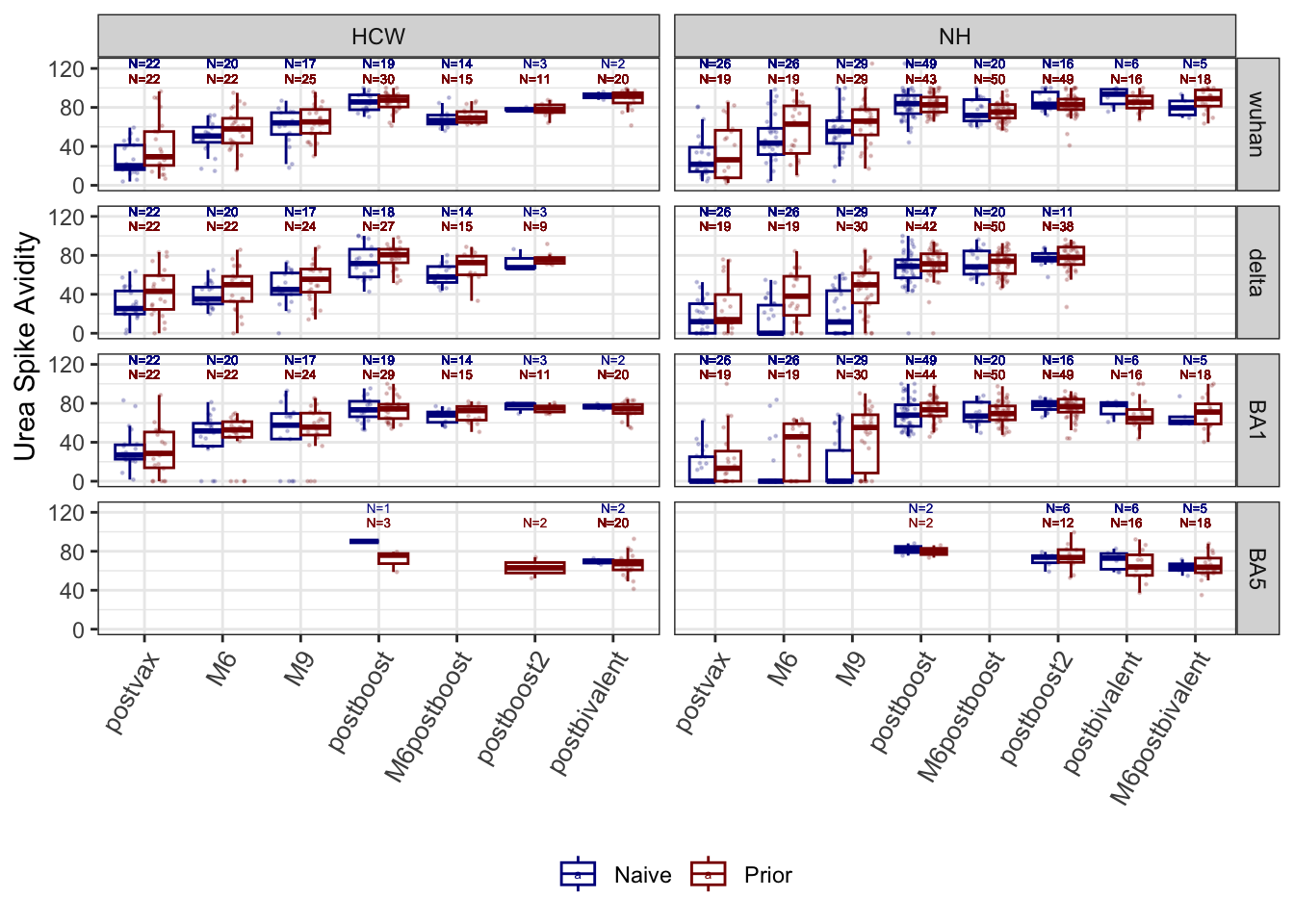


Fig. S3. **Anti-Receptor-Binding Domain (RBD) avidity over time against Wuhan, Delta, BA.1 and BA.5 strains - nursing home residents (NH) & healthcare workers (HCW).** The figure shows the kinetics of anti-RBD avidity against the Wuhan, Delta, BA.1 and BA.5 strains across different time points among nursing home residents and healthcare workers. Avidity is measured by ELISA using 6M urea and expressed as avidity index in percentage (%). Boxplots show medians (middle line), and third and first quartiles (boxes), while the whiskers display the minimum and maximum values. Post-vaccination sera were taken 2-4 weeks after each dose while M6 and M9 sera were taken 6-8 months and 7-10 months later, respectively. Blue: Naive subjects, Red: Prior subjects. BV: Wuhan-Omicron BA.5 Bivalent booster.


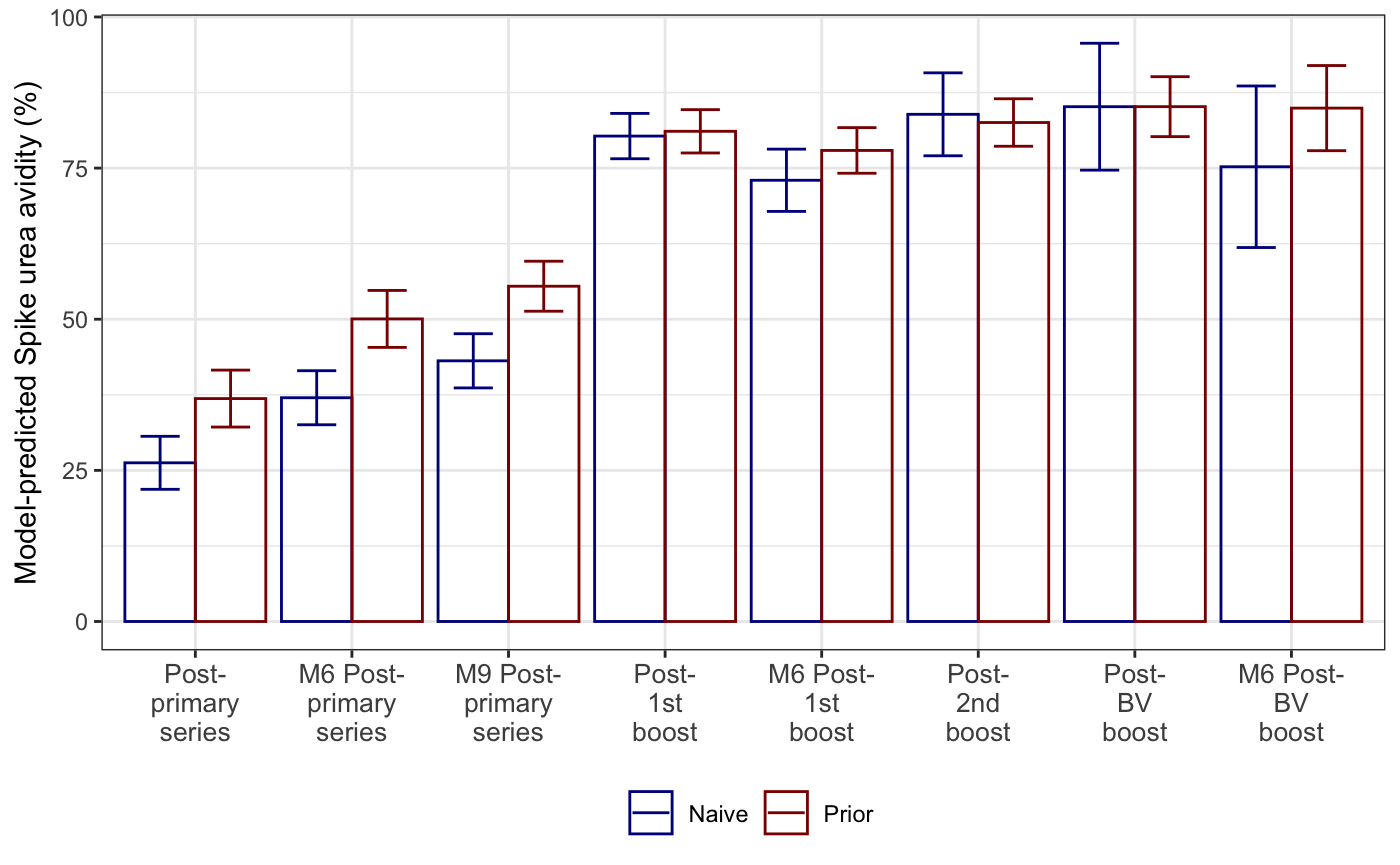
Fig. S4. **Model-predicted Wuhan Spike urea avidity.** A linear mixed-effects model was estimated to compare Wuhan urea avidity across time with possible interactions of cohort and infection status. No cohort effect was detected. Estimated marginal mean values with 95% confidence intervals by time and status are presented in Figure. With post-hoc contrasts comparing infection naive to prior infection at each time, avidity differed significantly (p < 0.05) for all pre-boost sampling times, while no differences were detected at any post-boost sampling times. Comparing paired sampling times within infection status, we found that all pre-boost sampling points differed from all post-boost sampling times (p < 0.05 for all) and no differences were detected among the post-boost sampling times, further demonstrating the plateau of avidity following doses beyond primary series. Post-vaccination sera were taken 2-4 weeks after each dose while M6 and M9 sera were taken 6-8 months and 7-10 months later, respectively. Blue: Naive subjects, Red: Prior subjects. BV: Wuhan-Omicron BA.5 Bivalent booster.


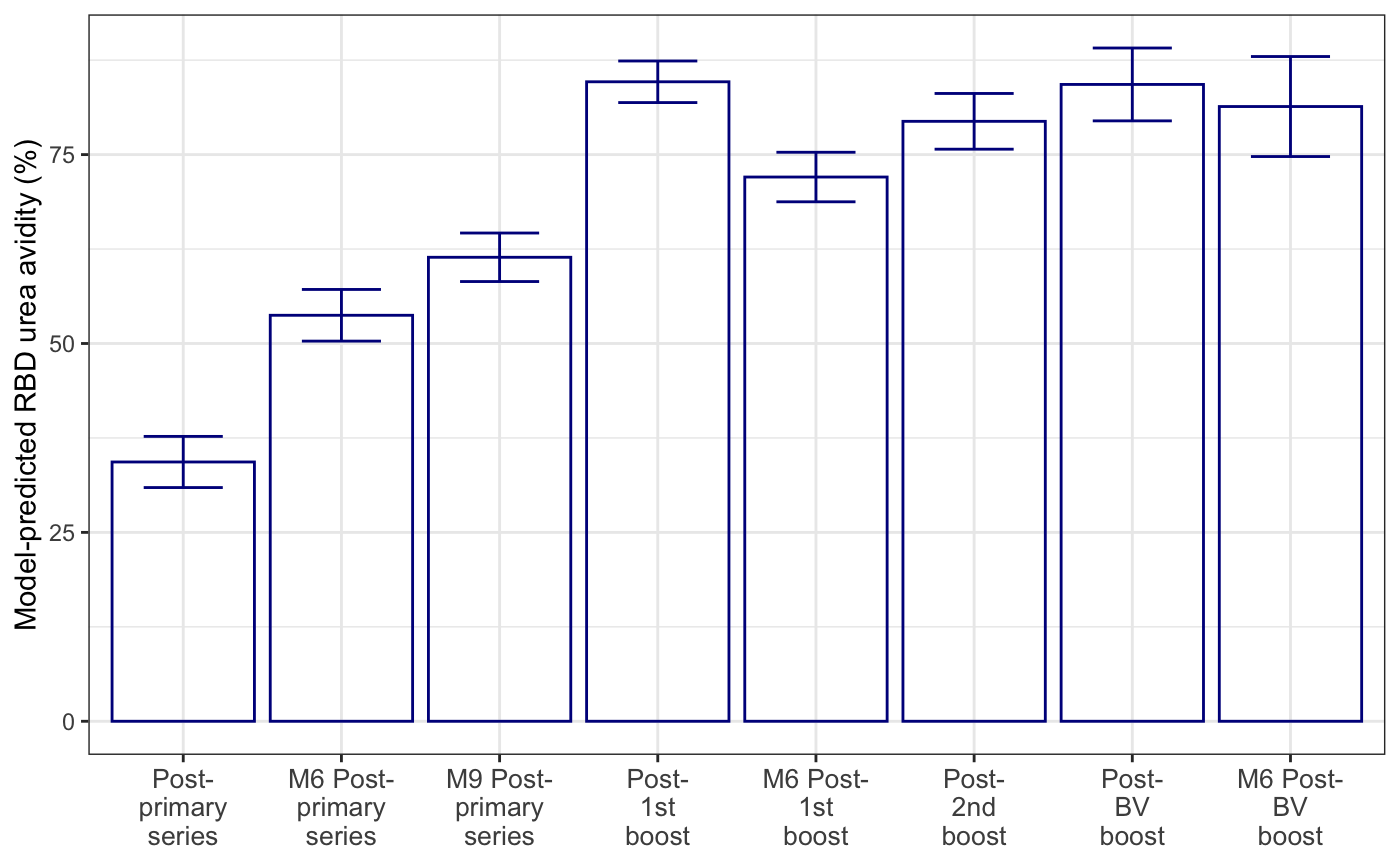


Fig. S5. **Model-predicted Wuhan RBD urea avidity.** A linear mixed-effects model was estimated to compare Wuhan urea avidity across time with possible interactions of cohort and infection status. No cohort effect was detected and no time-by-status interaction effect was detected. Estimated marginal mean values, averaged across status, with 95% confidence intervals by time, are presented in Figure. Comparing paired sampling times, all pre-boost sampling points differed from all post-boost sampling times (p < 0.05 for all), and, except for the M6 post-1st boost, no differences were detected among the post-boost sampling times. Post-vaccination sera were taken 2-4 weeks after each dose while M6 and M9 sera were taken 6-8 months and 7-10 months later, respectively. Blue: Naive subjects, Red: Prior subjects. BV: Wuhan-Omicron BA.5 Bivalent booster.
